# How to Avoid Choking

**Published:** 1891-09

**Authors:** 


### HOW TO AVOID CHOKING.

Death by the clogging of the windpipe is an accident liable to happen to hungry persons eating hastily, or to children, and calls for the greatest self control and presence of mind on the part of those who are present. The substance which causes the choking may either be at the top of the throat, at the entrance to the gullet, or lower down. If at the upper part of the throat, prompt action will often remove it, either by thrusting the finger and thumb into the mouth and pulling the obstruction away, or, if it cannot be reached so as to pull it away, a piece of whalebone, a quill, or even a penholder—any thing at hand—should be seized and pushed down as a probang, so as to force the substance down the gullet. Tickling the back of the mouth with a feather, so as to produce violent retching, will sometimes dislodge it, or a sudden splashing of cold water in the face, which causes involuntary gasping. Should the patient become insensible before relief can be afforded, it must not be assumed that death has taken place, and such remedies as dashing cold water in the face and on the chest, and applying ammonia to the nostrils should be continued till medical aid arrives.

